# Core Mental Health Data Set (CMHDS) methods feasibility paper

**DOI:** 10.1136/bmjhci-2025-101446

**Published:** 2025-12-12

**Authors:** Kathryn Mary Abel, Auden Edwardes, Heidi Tranter, Paul Dark, Robert D Sandler, Philip A Kalra, Ann John, Martin Wildman, Philip Bell, Nawar Diar Bakerly, Pauline Whelan

**Affiliations:** 1 Centre for Women’s Mental Health, The University of Manchester Faculty of Biology Medicine and Health, Manchester, England, UK; 2 GM.Digital Research Unit, Greater Manchester Mental Health NHS Foundation Trust, Manchester, UK; 3 Salford Care Organisation, Northern Care Alliance NHS Foundation Trust, Salford, England, UK; 4 Adult CF Centre, Sheffield Teaching Hospitals NHS Foundation Trust, Sheffield, UK; 5 Division of Population Health, The University of Sheffield, Sheffield, UK; 6 School of Health Data Science, Swansea University, Swansea, UK; 7 Royal Hallamshire Hospital, Sheffield Teaching Hospitals NHS Foundation Trust, Sheffield, England, UK; 8 Primer, The University of Manchester, Manchester, England, UK; 9 Oaklands Hospital, Salford, England, UK; 10 Euxton Hall Hospital, Chorley, England, UK; 11 Division of Informatics, Imaging and Data Sciences, The University of Manchester Faculty of Biology Medicine and Health, Manchester, England, UK

**Keywords:** Health Information Systems, BMJ Health Informatics, Patient Involvement, Data Systems

## Abstract

**Objectives:**

Little research focuses on mechanisms underlying the well-recognised relationship between mental and physical health, or its potential to influence adherence and response to treatments. This short report summarises results of the National Institute for Health and Care Research-funded ‘Core Mental Health Data Set (CMHDS)’ study to embed a digital tool for routine collection of mental health data in physical health studies.

**Methods:**

Four chief investigators of physical health trials were approached to embed the CMHDS into their study. Two trials, one for people receiving specialist cystic fibrosis (CF) care, and the established Salford Kidney Study (SKS) successfully managed to embed CMHDS.

**Results:**

A combined 478 participants from both studies were invited to complete the CMHDS. Of those approached, 88% agreed to complete CMHDS; 44% completed it. In the SKS, people who completed CMHDS were significantly younger and had higher estimated glomerular filtration rates and were from least deprived areas. In the CF study, there was no significant difference in characteristics of participants who did or did not complete the tool.

**Discussion:**

It was feasible, and researchers and participants considered it acceptable, to embed the CMHDS in physical health studies as part of routine data collection.

**Conclusion:**

Future studies should embed the CMHDS routinely and encourage completion to minimise bias and optimise the added value of having mental health covariates or predictor variables in physical health studies.

## Introduction

 The relationship between mental and physical health is well-recognised yet poorly understood. People with physical long-term conditions, such as ischaemic heart disease, cancer and autoimmune conditions, report higher rates of mental ill health than the general population.^1 2^ Little research examines mechanisms underlying this relationship, or whether/how mental health influences response to treatment. From 2022 to 2023, over 1 million people participated in National Institute for Health and Care Research (NIHR)-funded studies,^3^ of which >90% examined physical health conditions, yet fewer than 2% collected concurrent mental health data. Most clinical/cost-effectiveness studies collect quality of life measures, focussing on physical functioning and symptom burden, but do not reliably ascertain mental health status.^4^ For the first time, a novel co-developed digital tool, the Core Mental Health Data Set (CMHDS), collected mental health data in physical health studies.^5^ This study aimed to explore the feasibility of embedding CMHDS in physical health studies, not to capture links between physical and mental health.

## Methods

The CMHDS was co-developed with over 100 stakeholders and is a composite, digital questionnaire (see online supplemental appendix 1). Stepped consent within the CMHDS allowed participants to complete some or all questions. Feasibility was assessed and measured across three elements (online supplemental appendix 2). This was a convenience sample, with researchers contacting as many of the cohort as possible in the study period (online supplemental appendix 3). Of those contacted, 139 were spoken to; 61 did not respond and were not counted.

## Results

Four chief investigators agreed to embed the CMHDS; only the cystic fibrosis (CF) and Salford Kidney Study (SKS) studies did so. 478 participants across both studies were invited to complete the CMHDS. Of those, 88% agreed to complete it, and 44% ultimately completed it. Response rates were lower in the CF study; all participants were approached via email, with two email reminders for non-responders (online supplemental appendix 4).

In the SKS, participants who consented to and completed the CMHDS were significantly younger (median age 62 (IQR 54–74) vs 71 (61–80) years, p<0.001), had higher renal function (estimated glomerular filtration rate 34 (20–61) vs 29 (18–42) mL/min, p=0.049) and were more likely to be from higher socioeconomic groups. 56% of responders were in index of multiple deprivation (IMD) quintiles 4 or 5, compared with 29% of non-respondents, of whom almost half were in the lowest IMD quintile. In the CF study, there were no significant differences in participant characteristics between respondents and non-respondents (online supplemental appendix 5).

## Discussion

It was acceptable to researchers, staff and participants to embed the CMHDS. This has potential to improve knowledge regarding the influence of mental health on uptake and outcomes of physical health interventions. Age and socioeconomic status appeared to influence completion of the CMHDS in our study. Older individuals may access the internet less frequently, and therefore may be less likely to complete online questionnaires.^6^ Additionally, individuals from more deprived areas have higher rates of severe mental illness and may experience digital exclusion, so may be also less likely to complete the CMHDS. Therefore, we expect younger adults from less deprived areas to be more likely to engage with the CMHDS, as reflected in the respondent characteristics. The convenience sampling method may have led to under-representation of those with more severe mental illness from more deprived areas.

Future work should address perceptions regarding the importance of mental health data collection for improving health; clarifying how data are accessed and used to improve outcomes, especially for those in lower socioeconomic groups. Embedding the CMHDS in high-throughput routine settings such as acute care triage or general practice is also likely to optimise uptake. The DATAMIND hub^7^ will take this work forward, including cultural adaptation of the CMHDS to improve completion rates. Mandating the CMHDS for use in NIHR and Medical Research Council funded physical health studies would grow the data value in Department of Health and Social Care funded clinical research and provide a unique, low-cost, high value resource to understand mechanisms linking mental and physical health.

## Supplementary material

10.1136/bmjhci-2025-101446 online supplemental appendix 1

10.1136/bmjhci-2025-101446 online supplemental appendix 2

10.1136/bmjhci-2025-101446 online supplemental appendix 3

10.1136/bmjhci-2025-101446 online supplemental appendix 4

10.1136/bmjhci-2025-101446 online supplemental appendix 5
