# Supplementary material for: Core Mental Health Data Set (CMHDS) methods feasibility paper
Source: BMJ Health Care Inform. 2025 Dec 12;32(1):e101446. doi: 10.1136/bmjhci-2025-101446 (PMC12699609; doi:10.1136/bmjhci-2025-101446)
Supplement: online supplemental appendix 1 [file bmjhci-32-1-s001.docx]

Appendix 1 – CMHDS Full Questionnaire

**General Health Questionnaire**

This is questionnaire 1 of 5.

The first questionnaire is the Short General Health Questionnaire. This questionnaire includes questions about you recent well-being.

The questionnaire asks about things such as your sleep, concentration, happiness, and decision making.

If you wish to take this questionnaire, please click 'yes', or if you do not wish to take this questionnaire, please click 'no'.'

If you do not want to take any more questionnaires, please click 'exit.

 If you are feeling upset, please click the 'Help and Advice' button.

**Take this survey?**

Yes (if selected, the participant is shown the questions below)

No (if selected, the questionnaire moves on to the next questionnaire)

Have you recently?

| Been able to concentrate on what you’re doing? | Better than usual | Same as usual | Less so than usual | Much less than usual |
| --- | --- | --- | --- | --- |
| Lost much sleep over worry? | Not at all | No more than usual | Rather more than usual | Much more than usual |
| Felt you were playing a useful part in things? | More so than usual | Same as usual | Less useful than usual | Much less than usual |
| Felt capable of making decisions about things? | More so than usual | Same as usual | Less so than usual | Much less capable |
| Felt constantly under strain? | Not at all | No more than usual | Rather more than usual | Much more than usual |
| Felt you couldn’t overcome your difficulties? | Not at all | No more than usual | Rather more than usual | Much more than usual |
| Been able to enjoy your normal day-to-day activities? | More so than usual | Same as usual | Less so than usual | Much less than usual |
| Been able to face up to your problems? | More so than usual | Same as usual | Less so than usual | Much less than usual |
| Been feeling unhappy and depressed? | Not at all | No more than usual | Rather more than usual | Much more than usual |
| Been losing confidence in yourself? | Not at all | No more than usual | Rather more than usual | Much more than usual |
| Been thinking of yourself as a worthless person? | Not at all | No more than usual | Rather more than usual | Much more than usual |
| Been feeling reasonably happy, all things considered? | More so than usual | About same as usual | Less so than usual | Much less than usual |


**Mental Health History Questionnaire**

This is questionnaire 2 of 5.

The next questionnaire we would like you to complete is a mental health history questionnaire.

This questionnaire is designed to see if you have received a mental health diagnosis in the past or have sought treatment for a mental health condition. We would like you to complete the questionnaire even if you have never received a mental health diagnosis or sought treatment.

If you wish to take this questionnaire, please click 'yes', or if you do not wish to take this questionnaire, please click 'no'.'

If you do not want to take any more questionnaires, please click 'exit.

 If you are feeling upset, please click the 'Help and Advice' button.

**Take this questionnaire?**

**Have you been diagnosed with one or more of the following mental health problems by a professional, even if you don't have it currently (tick all that apply):**

**By a professional we mean: any doctor, nurse or person with specialist training (such as a psychologist or therapist). Please include disorders even if you did not need treatment for them or if you did not agree with the diagnosis.**

**Depression**

The NHS describes depression as a low mood that lasts for weeks or months and affects your daily life.

**Mania, bipolar, hypomania, manic-depression**

The NHS describes this as having periods of depression (feeling very low and lethargic) and periods of mania (feeling very high and overactive). Unlike simple mood swings, each episode is extreme and can last for weeks or longer.

**Schizophrenia**

The NHS describes schizophrenia as a type of psychosis. This means the person may not always be able to distinguish their own thoughts and ideas from reality.

**Psychosis or any other psychotic illness**

The NHS describes this as an illness where you see or hear things that are not there (hallucinations) or believe things that are not true (delusions).

**Autism, Aspergers or ASD**

The NHS describes autism as a lifelong developmental disability, which affects how a person communicates, relates to other people and makes sense of the world around them.

Other terms for autism:

- autism spectrum disorder (ASD) is the medical name for autism
- Asperger's (or Asperger syndrome) is used by some people to describe autistic people with average or above average intelligence

**ADHD**

The NHS describes Attention deficit hyperactivity disorder (ADHD) as a condition that affects people's behaviour. People with ADHD can seem restless, may have trouble concentrating and may act on impulse.

**Anxiety**

The NHS defines anxiety is a feeling of unease, such as worry or fear, that can be mild or severe.

**Generalised Anxiety Disorder**

The NHS defines Generalised Anxiety Disorder (GAD) as a long-term condition that causes you to feel anxious about a wide range of situations and issues, rather than 1 specific event.

People with GAD feel anxious most days and often struggle to remember the last time they felt relaxed.

**Social Anxiety or Social Phobia**

The NHS describes this as a long-term and overwhelming fear of social situations. The NHS says this is more than social anxiety is more than shyness. It's a fear that does not go away and affects everyday activities, self confidence, relationships and work or school life. Many people occasionally worry about social situations, but someone with social anxiety feels overly worried before, during and after them.

**Panic Attacks**

The NHS describes a panic attack as when your body experiences a rush of intense psychological (mental) and physical symptoms. You may experience an overwhelming sense of fear, apprehension and anxiety.

**Obsessive Compulsive Disorder (OCD)**

The NHS describes Obsessive Compulsive Disorder (OCD) as a mental health condition. It causes a person to have obsessive thoughts and carry out compulsive activity. This can be distressing and can have a big impact on your life.

**Post Traumatic Stress Disorder (PTSD**)

The NHS describes Post-traumatic stress disorder (PTSD) as a mental health condition caused by very stressful, frightening or distressing events.

**Anorexia Nervosa**

The NHS describes this as an eating disorder and serious mental health condition, where people try to keep their weight as low as possible. They may do this in different ways, such as not eating enough food, exercising too much, taking laxatives or making themselves sick (vomit).

**Bulima Nervosa**

The NHS describes this as an eating disorder and mental health condition, where people go through periods where they eat a lot of food in a very short amount of time (binge eating) and then purge the food from their body to try to stop themselves gaining weight.

**Binge-eating Disorder**

The NHS describes this as regularly eating a lot of food over a short period of time until you're uncomfortably full.

Any other type of psychosis or psychotic illness

Any other phobia (e.g., disabling fear of heights or spiders)

Any other eating disorder

None of the above

**Have you accessed any of the following forms of support in the past for your mental health?**

Medication

Individual therapy

Group therapy

Self-help apps

**Patient Health Questionnaire**

This is questionnaire 2 of 4.

The next questionnaire is the Patient Health Questionnaire. This questionnaire includes questions about your mood over the last 2 weeks.

The questionnaire asks about things such as your energy levels, appetite and feelings.

If you wish to take this questionnaire, please click 'yes', or if you do not wish to take this questionnaire, please click 'no'.'

If you do not want to take any more questionnaires, please click 'exit.

 If you are feeling upset, please click the 'Help and Advice' button.

**Over the last 2 weeks, how often have you been bothered by any of the following problems?**

|  | Not at all | Several Days | More than half the days | Nearly every day |
| --- | --- | --- | --- | --- |
| Little interest or pleasure in doing things | 0 | 1 | 2 | 3 |
| Feeling down, depressed, or hopeless | 0 | 1 | 2 | 3 |
| Trouble falling or staying asleep or sleeping too much. | 0 | 1 | 2 | 3 |
| Feeling tired or having little energy | 0 | 1 | 2 | 3 |
| Poor appetite or overeating | 0 | 1 | 2 | 3 |
| Feeling bad about yourself, or that you are a failure, or have let yourself or your family down | 0 | 1 | 2 | 3 |
| Trouble concentrating on things, such as reading the newspaper or watching television | 0 | 1 | 2 | 3 |
| Moving or speaking so slowly that other people could have noticed. Or the opposite – being so fidgety or restless that you have been moving around a lot more than usual | 0 | 1 | 2 | 3 |
| Thoughts that you would be better off dead or of hurting yourself in some way | 0 | 1 | 2 | 3 |

**Generalised Anxiety Disorder Assessment**

This is questionnaire 4 of 5.

The next questionnaire is the Generalised Anxiety Disorder Assessment. This questionnaire includes questions about your anxiety in the last 2 weeks.

The questionnaire asks about things such as worry, irritability and nervousness.

If you wish to take this questionnaire, please click 'yes', or if you do not wish to take this questionnaire, please click 'no'.'

If you do not want to take any more questionnaires, please click 'exit.

 If you are feeling upset, please click the 'Help and Advice' button.

**General Anxiety Disorder 7-item (GAD-7)**

Over the last 2 weeks, how often have you been bothered by any of the following problems?

|  | Not at all | Several Days | Over half the days | Nearly every day |
| --- | --- | --- | --- | --- |
| Feeling nervous, anxious, or on edge | 0 | 1 | 2 | 3 |
| Not being able to stop or control worrying | 0 | 1 | 2 | 3 |
| Worrying too much about different things | 0 | 1 | 2 | 3 |
| Trouble relaxing | 0 | 1 | 2 | 3 |
| Being so restless that it's hard to sit still | 0 | 1 | 2 | 3 |
| Becoming easily annoyed or irritable | 0 | 1 | 2 | 3 |
| Feeling afraid as if something awful might happen | 0 | 1 | 2 | 3 |
| *Add the score for each column* |  |  |  |  |

Total Score (add your column scores) =

If you checked off any problems, how difficult have these made it for you to do your work, take care of things at home, or get along with other people? (Circle one)

Not difficult at all Somewhat difficult Very difficult Extremely difficult

**Trauma Question**

This is questionnaire 5 of 5.

The next questionnaire is the Trauma Question.

Trauma refers to any distressing or upsetting experiences.

**If you think you will find thinking about past trauma distressing, you can choose not to complete this questionnaire by clicking 'no'.**

It does not ask you to share details about the specific trauma you have experienced.

If you have experienced trauma and want to seek help, please go to the seeking support tab in the top right-hand corner.

If you wish to take this questionnaire, please click 'yes', or if you do not wish to take this questionnaire, please click 'no'. If you do not want to take any more questionnaires, please click 'exit'

**Are there any traumatic events that you feel are relevant to your mental health?**

Yes, childhood trauma

Yes, other recent trauma

No relevant trauma
