# Supplementary material for: Core Mental Health Data Set (CMHDS) methods feasibility paper
Source: BMJ Health Care Inform. 2025 Dec 12;32(1):e101446. doi: 10.1136/bmjhci-2025-101446 (PMC12699609; doi:10.1136/bmjhci-2025-101446)
Supplement: online supplemental appendix 2 [file bmjhci-32-1-s002.docx]

**Appendix 1**

**CMHDS Feasibility Elements**

1. *Acceptability to researchers of embedding CMHDS.*
   - Proportion of Chief Investigators (CIs) approached, who agreed to embed the CMHDS in their study.
   - Four physical health study CIs were approached to embed the CMHDS. Two studies were able to implement the CMHDS, the CF study and SKS.
2. *Acceptability of CMHDS to participants.*
   - Proportion of participants approached who agreed to complete CMHDS.
3. *Recruitment of trial participants into CMHDS.*
   - Proportion of participants who ultimately completed the CMHDS after agreeing to do so.
