# Supplementary material for: Core Mental Health Data Set (CMHDS) methods feasibility paper
Source: BMJ Health Care Inform. 2025 Dec 12;32(1):e101446. doi: 10.1136/bmjhci-2025-101446 (PMC12699609; doi:10.1136/bmjhci-2025-101446)
Supplement: online supplemental appendix 3 [file bmjhci-32-1-s003.docx]

**Appendix 2**

**Recruitment methods**

Everyone receiving Cystic Fibrosis (CF) care at a UK adult CF Centre was invited to participate by email (n=227). New participants recruited to the Salford Kidney Study (SKS) cohort from August 2022-May 2023 were invited to participate in person or by phone (n=112). Existing SKS-cohort participants were asked to complete CMHDS (n=200) via email. The CMHDS was integrated into each study through participants being sent a link to complete the CMHDS by each study team. Data from the CMHDS was stored within each study in line with ethical approvals.
