# Supplementary figures and images for: Core Mental Health Data Set (CMHDS) methods feasibility paper
Source: BMJ Health Care Inform. 2025 Dec 12;32(1):e101446. doi: 10.1136/bmjhci-2025-101446 (PMC12699609; doi:10.1136/bmjhci-2025-101446)

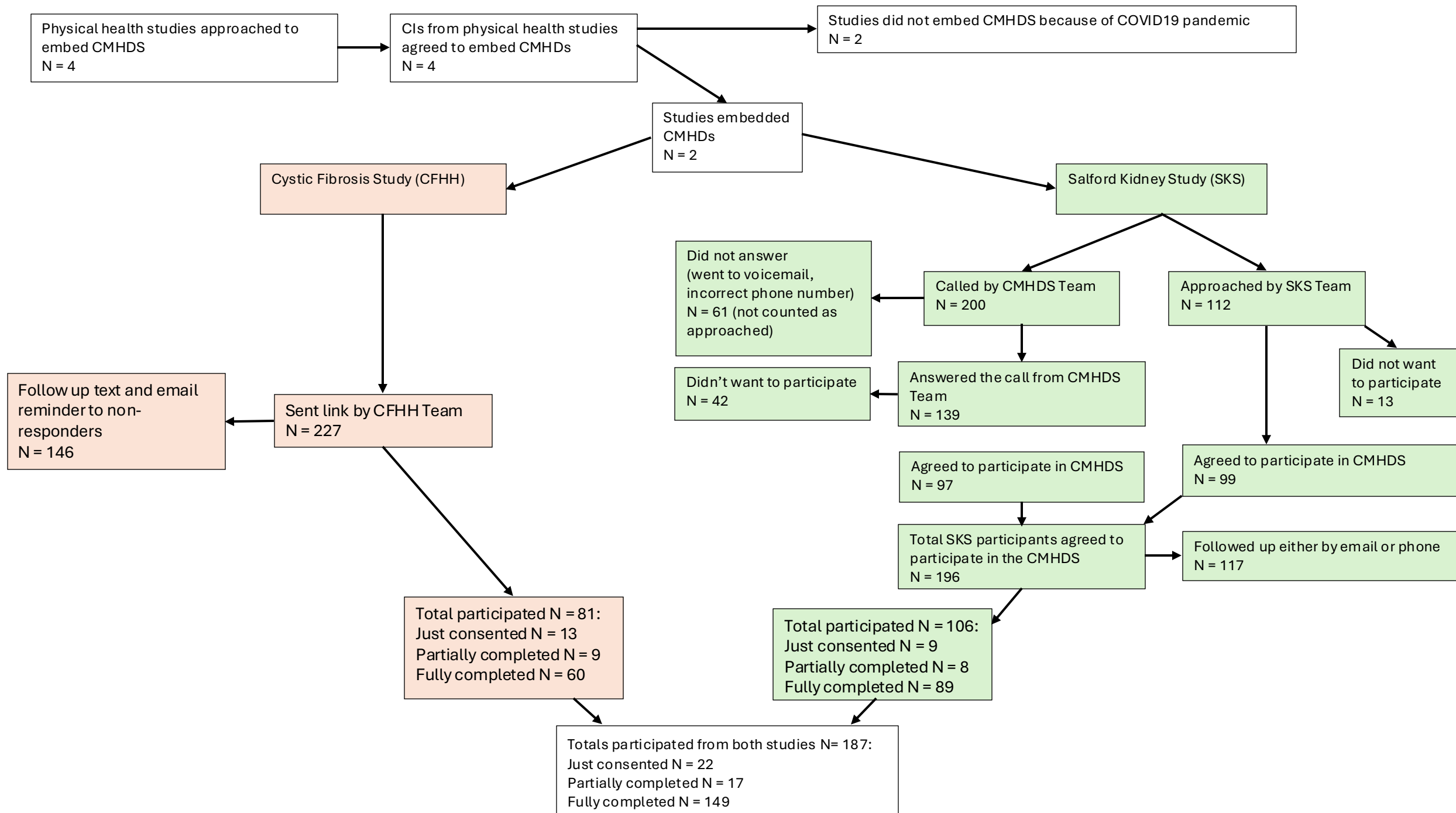

Supplement: online supplemental appendix 4 [file bmjhci-32-1-s004.pdf]
