# Supplementary material for: Core Mental Health Data Set (CMHDS) methods feasibility paper
Source: BMJ Health Care Inform. 2025 Dec 12;32(1):e101446. doi: 10.1136/bmjhci-2025-101446 (PMC12699609; doi:10.1136/bmjhci-2025-101446)
Supplement: online supplemental appendix 5 [file bmjhci-32-1-s005.docx]

**Appendix 4**

**Recruitment and participation data**

| **Study** | **N participants spoken to about completing the CMHDS** | **N (%) participants who were spoken to and agreed to be sent the CMHDS** | **N (%) participants who consented to and completed the CMHDS** |
| --- | --- | --- | --- |
| CF Study | 227 | 227 – sent to all | 81 (36%) |
| SKS | 251 | 196 (78%) | 106 (54%) |
| Total | 478 | 196 (78%) | 187 (44%) |

Most participants in SKS approached via telephone agreed to be sent the CMHDS, with over half completing it. Detailed participant characteristics of SKS participants who consented to and completed the CMHDS are as follows:

- Younger (median age 62 [IQR 54–74] vs 71 [61–80] years, p<0.001)
- Higher renal function (eGFR 34 [20–61] vs 29 [18–42] ml/min, p=0.049)
- More likely to be from higher socioeconomic groups, 56% of responders were in IMD quintiles 4 or 5, compared to 29% of non-respondents, with almost half of these in the lowest IMD quintile.
